# Draft Genome Sequence of *Duganella* sp. Strain DN04, Isolated from Cultivated Soil

**DOI:** 10.1128/MRA.00848-19

**Published:** 2019-08-08

**Authors:** Rachel Raths, Vincent Peta, Heike Bücking

**Affiliations:** a Biology and Microbiology Department, South Dakota State University, Brookings, South Dakota, USA; University of Delaware

## Abstract

Here, we sequenced *Duganella* sp. strain DN04, a novel species within the genus Duganella that was isolated from a maize field in North Carolina. The assembled draft genome size is 6,562,230 bp, with a total of 6,039 protein coding sequences and 3,889 functionally assigned genes, including genes putatively involved in the colonization of plants.

## ANNOUNCEMENT

The genus Duganella within the family Oxalobacteraceae was identified in 1997 (1) and consists of Gram-negative, motile, aerobic, mesophilic bacteria that are mainly found in soils. Most *Duganella* strains produce the bis-indole pigment violacein, which has antifungal and antibacterial properties (2, 3). Other *Duganella* strains show proteolytic and lipolytic activities (4).

*Duganella* sp. strain DN04 was isolated from a maize field in North Carolina on 9 June 2016 (geographical coordinates, 36.1034, −78.4114; soil pH, 5.8). Soil was added to phosphate-buffered saline, and dilutions were plated on Reasoner’s 2A (R2A) agar plates and incubated at 30°C for 1 to 2 days, followed by 20°C for 1 to 2 days. Repeated colony transfers and streaks were performed on R2A agar to acquire pure colonies. Genomic DNA was extracted from a freshly grown R2A culture using the AllPrep bacterial DNA/RNA/protein kit (Qiagen, Inc., Germantown, MD) following the kit protocol. A genomic library was prepared using a Nextera kit (Illumina, San Diego, CA), size selected to an average fragment length of 475 bp, and sequenced using Illumina NextSeq paired-end v2 chemistry on v2.5 flow cells at 150 bp per read. A target coverage of 20× was used, and the genome was assembled using SPAdes v3.13.0 (5). Default parameters were used for all software unless otherwise specified. The genome was screened for possible contamination by a BLAST search of annotated coding regions that represented at least 60% of the contigs against a diverse set of genomes. If less than 10% of the hits were to proteins from other species in the same family, the contamination was considered to be low.

A total of 11,788,390 reads were obtained, with a total read length of 1,738,895,421 bp and an average read length of 148 bp. The genome length was 6,562,230 bp. In total, we found 281 contigs with an *N*_50_ value of 40,161 bp (range, 671 to 141,855 bp) and an *L*_50_ value of 49. Assembly quality assessment using Benchmarking Universal Single-Copy Orthologs (BUSCO) (6) revealed a measured completeness (40 single-copy BUSCOs) of 100%. Following genome annotation by PATRIC v3.5.28 (7), we identified a total of 6,039 protein coding sequences with 2,150 hypothetical genes and 3,889 genes with functional assignments. Strain DN04 has 48 tRNA genes, 4 rRNA genes, 43 antibiotic resistance genes, and a GC content of 64.4%. Based on 16S rRNA, Duganella sacchari strain Sac-22 is the closest related species (98.82% identity).

Galaxy and RAST v2.0 were used to annotate and identify specific genes (8, 9). We discovered several genes that are putatively involved in plant growth promotion, including genes needed to catalyze the decomposition of hydrogen peroxide (*katE*) and genes involved in the production of urease (*ureA* to *ureG*), biofilms (*bdcA* and *wspC*) (10), and biotin (*bioA* to *bioD* and *bioF*) (11), several genes of the *pst* operon, and a two-component signal transduction system involved in phosphate uptake (*phoR*, *phoB*, *phoD*, *pstS*, *pstC*, *pstA*, *pstB*, *phoU*, and *ppk*) (12). We also identified 60 putative virulence genes, including 45 antibiotic and toxic compound resistance genes and 15 invasion and intracellular resistance genes.

### Data availability.

The complete genome sequence described here has been deposited in NCBI/GenBank under BioProject number PRJNA529278, BioSample number SAMN11263562, accession number NZ_SPVG00000000, and SRA number SRX6098754.
